# Effects of Problem-Based Learning on Critical Thinking, Communication Skills, and Satisfaction Among First-Year Medical Students in Ghana. A study protocol

**DOI:** 10.12688/mep.21103.1

**Published:** 2025-07-09

**Authors:** Bruce Ayabilla Abugri, Maxwell Ateni Assibi, Anthony Amalba, Patience Kanyiri Gaa, Sophia E. A. Kpebu, Victor Mogre

**Affiliations:** 1 Department of Health Professions Education and Innovative Learning, School of Medicine Professions Education and Innovative Learning, University for Development Studies, Tamale, Northern Region, 00233, Ghana; 2 Department of Pharmaceutical Practice, School of Pharmacy and Pharmaceutical Sciences, University for Development Studies, Tamale, Northern Region, 00233, Ghana; 3 Department of Dietetics, School of Allied Health Science, University for Development Studies, Tamale, Northern Region, 00233, Ghana

**Keywords:** Problem-based learning, critical thinking, communication skills, student satisfaction, medical education, Ghana

## Abstract

**Background:**

Problem-Based Learning (PBL) is increasingly used in medical education to develop critical thinking, communication, and learner autonomy. Although widely studied in developed countries, evidence from low-resource settings like Ghana, particularly involving first-year students, remains limited. This study seeks to address this gap by assessing the impact of PBL on critical thinking, communication skills, and satisfaction levels among novice medical students.

**Methods:**

This longitudinal pre-test-post-test study will be conducted at the University for Development Studies, School of Medicine. All first-year students who meet the eligibility criteria will be enrolled. Participants will undergo baseline (pre-PBL) and follow-up (post-PBL) assessments using the Critical Thinking Questionnaire (licensed under a Creative Commons Attribution 4.0 International License (CC BY 4.0) and Interpersonal Communication Competency Skills Scale (ICCS). A 5-point Likert scale will be used to measure student satisfaction after eight months of exposure to PBL. Statistical analyses including paired t-tests, ANOVA, and regression will be performed using SPSS v26.0. to assess changes through the mean scores.

**Discussion:**

The study will provide context-specific insights on the effectiveness of PBL in enhancing key competencies among first-year medical students. Findings will inform curriculum development, tutor training, and educational policy in Ghana and similar settings.

**Clinical trial registration number:**

Not applicable.

## Introduction

### Background

Medical education is systematic in nature, but it changes over time to provide learners with the tools and skills necessary for effective patient care. Harden (2020) includes schooling or medical school followed by postgraduate education, which includes residency and specialist training, as well as continuing professional development or lifelong learning to accommodate any developments in the field of medicine. In an effort to advance clinical practice as well as theoretical knowledge, competency-based education (CBE), problem-based learning (PBL), and simulation-based training are some of the pedagogical designs that have been incorporated into medical education over time ( Frank *et al*., 2010).

Medical education was traditionally centred on apprenticeships, with a working physician teaching the art. However, a uniform curriculum founded on scientific rigor was developed with the Flexner Report in 1910, which revolutionized medical education both domestically and internationally ( Cooke *et al*., 2010). Although the core biological sciences were the focus of this paradigm, student-centred learning has gradually taken over in the decades that have followed, with a greater focus on communication, clinical reasoning, and critical thinking ( Schuwirth & van der Vleuten, 2020).

The twenty-first century brought revolutionary changes in the education of medicine, which included the use of technology-supported learning, competency-based examination, and interprofessional practice. Computer-aided instruction and virtual simulation are two types of digital training tools that have enhanced clinical skills acquisition and extended training access ( Ellaway & Masters, 2008). Focusing on demonstrated competence rather than time-based training, competency-based medical education has further progressed assessment tools ( Frank *et al*., 2010).

Problem-based learning (PBL) is one of the most suitable pedagogies since it encourages critical thinking, active learning, and communication skills, which are all essential in modern-day medical practice ( Neville, 2009). PBL prepares graduates for handling complicated medical cases by subjecting them to real-life clinical problems. This improves self-directed learning, teamwork, and problem-solving skills ( Dolmans *et al*., 2016). PBL is being used increasingly in many different learning environments and has become a key approach to enhancing educational outcomes in contemporary medical education.

The 1969 birth of problem-based learning (PBL) was among the greatest of innovations in the last half century of medical education. PBL has since been embraced by different learning settings in nearly all health professions' education systems ( Lim, 2023).

Decades ago, medical education faced challenges such as underdevelopment and inadequacy in preparing medical professionals to meet the evolving demands of patient care, health conditions, and healthcare systems. PBL continues to gain acceptance even when traditional learning didactic methods of teaching dominate ( More *et al*., 2020).

Acknowledging the pressing need for competency among medical professionals, a group of educators at McMaster University in Canada initiated the “McMaster Philosophy" project. This project aimed to revolutionize medical education by introducing innovative teaching and learning methods to produce competent and up-to-date medical practitioners ( Neufeld & Barrows, 1974). McMaster philosophy was later formalized into problem-based learning (PBL), which has since become a cornerstone of modern medical education. Although several criticisms are raised by criticists, such as learning must be by lectures, students see PBL as a rite and are not able to learn all they need to know, self-directed criticism may lead to errors in learning, and some also claim that students are too immature to use PBL, incompetencies of PBL facilitators, PBL problems not always improved, and PBL content overlap with lectures and that the PBL curriculum is too resource- and time-intensive to run. However, evidence from research indicates that the benefits of PBL are overwhelming.

PBL uses problems as a starting point for acquiring new knowledge ( Vargas-Rodríguez *et al*., 2021). Barrows (1986) defines PBL as a learning approach that uses problems as the basis for learning, shifting the focus from a teacher-centered model to a student-centered model that emphasizes self-directed learning. A systematic review by Sayyah *et al*. (2017) indicated that employing PBL in medical education positively affects the academic achievement of undergraduate medical students. Similarly, a scoping review by Trullàs *et al*. (2022) noted that PBL is a promising teaching and learning approach for medical education; they predicted the likelihood of PBL enabling students to develop other competencies necessary in their professional practice other than the acquisition of knowledge.

Currently a common teaching pedagogy in medical education, PBL is praised for fostering critical thinking, problem-solving skills, and self-directed learning among students. Its implementation in Ghana at the University for Development Studies, School of Medicine, since 2007 and University of Cape Coast, School of Medicine, ( Amoako-Sakyi & Amonoo-Kuofi, 2015; Mogre & Amalba, 2015) represents a significant educational innovation adopted by the institution.

Although criticisms of students' immaturity for self-directed learning, resource intensity, and duplication with traditional curricula have been noted, and suggested that evidence supports the enhancement of critical thinking, problem-solving, and communication skills through PBL Sayyah *et al*. (2017); Vargas-Rodríguez *et al*. (2021). According to Vargas-Rodríguez *et al*. (2021), PBL develops competencies other than knowledge acquisition and has become a cornerstone for modern medical education. PBL has gained widespread acceptance because it is perceived to engage students in real-life problem-solving situations that enhance active learning through cooperation and reflection. For this reason, several curricula have adopted PBL as a pedagogy to increase medical students' readiness for both academic and professional settings. The resources for this teaching methodology are plentiful; however, there are some issues associated with it. Nonetheless, the evidence supporting PBL is pivotal with respect to the development of relevant medical skills for the education in question.

In conclusion, while PBL is respected for fostering analytical skills, problem solving, and interpersonal communication, it is not clear how effective PBL is for first-year medical students. Manuaba *et al*. (2022) indicated that PBL did not add any considerable benefits compared with traditional methods of instruction and the enhancement of critical thinking and self-regulated learning, claiming that contextual and implementation factors may be highly determinative. Other studies conducted by Ocheretnyuk *et al*. (2018) and Picton *et al*. (2022) highlighted the significance of communication skill on the part of first-year students as well as critical thinking in professional adaptation and identification and the capability of PBL in responding to these needs because it is a collaborative approach. What this implies is that the effect of PBL can be modulated by medical students' transitional difficulties based on professional identification. Due to this, this research aims to examine more closely the role of PBL in the development and achievement of critical thinking and communication skills of first-year medical students to further enhance the knowledge on the effectiveness of PBL in early medical education.

This study is based on a unified single frame of reference that applies constructivist theory of learning, self-directed learning theory, and social learning theory to investigate how PBL constructs first-year medical students' critical thinking and communication skills. Constructivist learning theory ( Piaget & Duckworth, 1970; Vygotsky, 1978) is centered on knowledge construction from experience and reflection, imitation and behavior change, and the principles of social interaction supportive of the collaborative inquiry features of which there are in PBL ( Hmelo-Silver, 2004). Self-directed learning theory ( Knowles, 1975) is supportive of independent learners, who are in charge of determining the learning needs, locating the resources, and evaluating the outcomes. Social learning theory ( Bandura, 1977) extends the notion of independence to incorporate observations and modelling from peer interactions, which are foundational to the group-based nature of PBL learning.

The conceptual framework identifies PBL as the independent variable influencing two key outcomes: critical thinking (analytical reasoning, evidence-based decision-making) and communication skills (teamwork, professional dialogue, and active listening).

They are mediated by three mediating processes, metacognition, social interaction, and cognitive engagement, which are essential mechanisms whereby PBL exerts its influence. Several confounding variables are also controlled, including prior learning records, multiple learning styles, facilitator competence, student motivation, and the classroom environment.

This theoretical framework informs the design of the study and analytic plan. The Critical Thinking Questionnaire (CThQ) ( Kobylarek *et al*., 2022), licensed under a Creative Commons Attribution 4.0 International License (CC BY 4.0) will be used to assessed Critical thinkings skills, Interpersonal Communication Competency Skills Scale will also be assessed through the Interpersonal Communication Competency Scale (ICCS) and satisfaction levels through a 5-point Likert scale. Although mediating processes cannot be directly measured, their influence is recognized to be at the core of the learning process. The model offers a synthesis and theory-based framework for determining the effectiveness of PBL in developing underpinning clinical reasoning, collaborative practice, and lifelong learning skills in medical training.

### Problem statement

Problem-based learning (PBL) is increasingly promoted in health professional education because of its potential to improve the critical thinking, communication, and group working skills needed in clinical judgement and professional practice ( Dolmans *et al*., 2016; Neville, 2009). Medical education has experienced an immense paradigm shift to student-centered instruction since PBL was introduced.

However, issues arise regarding its specific effectiveness among first-year medical students who are just being introduced to self-directed and collaborative learning environments. A scoping review was conducted to examine the literature concerning the effects of PBL on critical thinking and communication skills in first-year undergraduate medical students in high- and low-resource settings. Although the review confirmed the potential of PBL to enhance these skills, two significant gaps were identified. The first was a contextual gap: no African countries, such as Ghana, where education and health systems may be significantly different from high-resource contexts. The second was a conceptual gap: there was only one study ( Yadav *et al*., 2018) in Nepal that focused on first-year medical students. This study measured generic skills such as analytical thinking, collaborative practice, and active listening via a cross-sectional design and self-report questionnaire, precluding its depth and capacity to infer causality. These findings reinforce the need for context specific, methodologically robust research on the effects of PBL among first-year medical students in resource-constrained settings, critical thinking and communication skills development and student satisfaction.

First-year students commonly find it difficult to cope with PBL, including handling independent study, dealing with group work, and actively participating in peer discussion ( Manuaba *et al*., 2022). Such transition challenges are also exacerbated when there is poor faculty direction, poor physical facilities, and inadequate formal training in communication skills ( Mughal & Shaikh, 2018). Student satisfaction, which is highly related to engagement, motivation, and learning achievement, has also been inappropriately balanced in the literature. Exceedingly high levels of satisfaction with PBL's interactive and collaborative structure have been cited in some papers ( AlHaqwi *et al*., 2015; Errabti *et al*., 2024), whereas others voice alarms over time, alignment in assessment, and facilitator preparation ( Animaw & Asaminew, 2023; Barnawi *et al*., 2024; Mohammed *et al*., 2024).

These gaps, particularly in relation to first-year studies in various African contexts, raise important concerns regarding the pedagogical integrity and contextual relevance of PBL. Longitudinal research is crucial to better understand novice learners' engagement with PBL and how this method affects long-term critical thinking skills, communication abilities, and levels of satisfaction. The findings will form the basis of curricular refinements and help inform implementational practices across similarly constrained academic environments.

### Objectives and research questions

***General objective***

To determine the effects of PBL on critical thinking, communication skills, and satisfaction levels among first-year medical students.

***Specific objectives***

1. To assess changes in students’ critical thinking skills after exposure to PBL.
2. To examine the effect of PBL on students’ communication skills.
3. To assess students’ satisfaction with PBL-based instruction.

***Research questions***

1. What is the effect of PBL on critical thinking skills among first-year medical students at the UDS?
2. What is the effect of PBL on communication skills?
3. What is the level of student satisfaction with their experience with PBL?

### Significance of the study

This study provides essential empirical evidence on how problem-based learning (PBL) influences critical thinking, communication skills, and satisfaction levels among first-year medical students in a low-resource context. While PBL has been integrated into medical curricula worldwide, existing research has not adequately explored its effect at the foundational level of training. This gap is especially visible in two areas: contextual and knowledge-based.

The contextual gap refers to the absence of studies conducted in African settings, particularly Ghana, where the educational, cultural, and infrastructural realities differ significantly from those in which most PBL research has been conducted. A recent scoping review by the research team revealed that all included studies on the effects of PBL were based in Asia, with no representation from African countries. This spatial disparity limits the generalizability of current findings to universities such as the University for Development Studies (UDS), which are plagued by problems such as larger class sizes, limited access to online resources, and fewer trained facilitators ( Jdaitawi, 2020; Mughal & Shaikh, 2018). The UDS, as one of the pioneers of Ghanaian universities to implement PBL ( Amoako-Sakyi & Amonoo-Kuofi, 2015; Mogre & Amalba, 2015), presents a suitable context in which to investigate how the approach performs in real-world, resource-limited environments.

The knowledge deficit represents the lack of evidence regarding the effect of PBL among first-year medical students in terms of critical thinking and communication skills development and students’ satisfaction. While various studies have revealed improved critical thinking and problem-solving among medical students taught via PBL ( Asad *et al*., 2015; Pu *et al*., 2019), virtually all of these studies involve upper-level students or combined samples that are not conducive to the first-year learner effect.

Only one study in the reviewed literature focused exclusively on first-year medical students and employed a descriptive, cross-sectional approach based solely on self-reported perceptions, making it difficult to establish any measurable change over time ( Yadav *et al*., 2018).

This study addresses both gaps through a quantitative pre-test‒post-test design that measures self-perceived changes in critical thinking and communication skills, as well as levels of satisfaction following PBL exposure. Unlike previous studies that relied on single-point feedback evidence, this research applies validated instruments to capture changes over time. Measuring outcomes in this way provides a more objective assessment of PBL’s effect on key educational competencies.

Critical thinking is a fundamental skill in medical education that influences diagnostic reasoning, clinical decision-making, and lifelong learning ( Ennis, 2018; Facione, 2011). PBL is widely believed to support its development by engaging students in inquiry, reflection, and peer interaction ( Dolmans *et al*., 2016; Neville, 2009). However, little is known about how effectively PBL fosters critical thinking in first-year students who may not yet have the foundational scientific knowledge or the metacognitive skills necessary to fully benefit from self-directed learning.

Similarly, communication skills are essential. Good and respectful interaction with patients, colleagues, and healthcare teams depends on a student’s ability to listen, express ideas clearly, and collaborate within interdisciplinary settings ( Kurtz *et al*., 2017; Silverman *et al*., 2016). PBL introduces students to group discussions, case presentations, and role-based learning, all of which can enhance communication competence. However, most available studies focus on later stages of training or are qualitative in nature ( Hande *et al*., 2015), offering limited insight into the early development of communication skills through PBL among new medical students.

Learner satisfaction also plays a key role in academic performance, motivation, and course engagement. Medical students’ perceptions of PBL vary widely, and dissatisfaction may stem from workload demands, a lack of clarity in learning outcomes, or weak tutor facilitation ( AlHaqwi *et al*., 2015; Errabti *et al*., 2024; Nahar *et al*., 2014). Understanding satisfaction levels through quantitative analysis provides measurable data that can inform improvements in PBL delivery and tutor training, particularly at the introductory level of medical education.

At the institutional level, the findings will enable the School of Medicine at the UDS to evaluate how well PBL is serving its intended goals. This includes examining whether students are acquiring the competencies the curriculum is designed to promote and whether they feel supported in the learning process. The evidence from this study will help guide decisions about resource allocation, facilitator development, and potential adjustments in how PBL is structured or introduced during the first year.

Other medical schools in Ghana and similar resource-limited contexts may also benefit from the findings. Many of these institutions are in the early stages of adopting PBL and often lack local data to inform implementation strategies. The use of context-relevant evidence from UDSs can provide a reference point for best practices or identify areas requiring institutional adaptation.

This research also contributes to broader policy discussions within medical education reform. Competency-based education models, which are increasingly endorsed at the regional and global levels, emphasize measurable outcomes and skill development over content delivery alone ( Frank *et al*., 2010). PBL is frequently presented as a vehicle for this transition. However, its effectiveness must be validated across diverse contexts through systematic, outcome-based research. Findings from this study will help build that evidence base, offering a clearer picture of how PBL performs in underrepresented regions such as sub-Saharan Africa.

In summary, this study is pertinent both in its setting and methodological contribution. This study provides measurable data on the effects of PBL on critical thinking, communication skills, and student satisfaction among first-year medical students at the UDS. This study addresses obvious gaps in the literature, strengthens evidence-based curriculum innovation, and contributes to the local and regional understanding of PBL's contribution to medical education. The results could enhance teaching and learning not only at the UDS but also at other universities experiencing the same learning and resource constraints.

## Methodology

### Study design

This research uses a quantitative longitudinal pretest and post-test design to evaluate the effect of problem-based learning (PBL) on critical thinking, communication skills, and satisfaction among first-year medical students at the University for Development Studies, School of Medicine (UDS-SoM), Tamale. The design allows for measurement of change at the individual level over time and is appropriate for the evaluation of the causal effect of education interventions via standard instruments ( Creswell & Creswell, 2017).

### Study setting

The study will be conducted at the UDS-SoM, which offers a PBL curriculum. The school is affiliated with the Tamale Teaching Hospital for the clinical training.

### Participants and inclusion criteria

***Target population***

All first-year medical students enrolled in the 2024–2025 academic year at UDS-SoM.

They are selected because they are fresh from their premedical school training and are not yet to be exposed to PBL in this institutional context.

***Inclusion criteria***

Enrolled as first-year medical students at the UDS-SoM during the study period

The participants will be first-year medical students aged between 18 and above.

Voluntary participation and willingness to provide informed consent

No prior exposure to PBL at any learning institution

In student screening for prior experience with PBL, a screening questionnaire will be given during the process of providing consent. The identified students will be excluded for the purpose of maintaining internal validity and for ensuring that the study assesses the effect of the UDS's implementation of PBL specifically.

### Sampling strategy and sample size

A census sampling approach will be adopted, inviting all potentially eligible first-year students to participate. This approach reduces selection bias and offers maximum representation of the cohort ( Etikan *et al*., 2016). Although there is no a priori sample size estimation needed with this approach, the historic enrolment records available show an estimated sample of approximately 100 students, which is considered sufficient to detect medium-to-large effect sizes in paired-sample tests ( Cohen, 2013).

### Intervention

Students will receive two times weekly, PBL sessions guided by facilitator for eight-month. Each session is based on case scenarios prepared from basic medical sciences that form the foundation of medicine. Students define learning objectives, learn independently, and return to share and discuss outcomes in groups. Facilitators foster questioning and group collaboration without providing direct solutions, encouraging autonomy and collaboration.

### Data collection instruments

The study will utilize three standard instruments: the Critical Thinking Questionnaire (CThQ), the Interpersonal Communication Competency Scale (ICCS) and a 5-point Likert scale. The CThQ examines critical thinking skills, the ICCS examines communication competency, and the 5-point Likert scale examines the students' satisfaction level. All the instruments have been used in previous research and are widely used in education studies ( Kobylarek *et al*., 2022; Rubin & Martin, 1994).

The CThQ is a reliable and valid tool for assessing critical thinking skills, with a Cronbach's alpha over 0.84 indicating that the tool is reliable enough. The questionnaire possesses a number of items for assessing critical thinking in various dimensions, such as remembering, understanding, applying, analysing, evaluating, and creating. The ICCS is a 30-item measure consisting of five subscales of interpersonal communication competence: self-disclosure, empathy, social relaxation, assertiveness, and altercentrism. A pilot study was utilized in developing the reliability of the scales, and a Cronbach's alpha of 0.84 and above was deemed acceptable reliability. Face and content validity will be determined by expert and participant ratings to determine whether the instruments are suitable for the task. The scales are piloted repeatedly to ensure that they are internally consistent and capable of measuring the constructs under investigation.

### Data collection procedure

Once ethical approval is acquired from the University for Development Studies Ethical Review Board, permission to proceed with the study will be sought from the University for Development Studies School of Medicine authorities. This is necessary to ensure that the study is in line with institutional policy and ethical practice ( Creswell & Creswell, 2017). Information sessions will be organized to inform the students about the purpose of the study, the procedures followed, and the rights of the participants. Such sessions are required to ensure transparency and to acquire informed consent, which is the basis of ethical research ( Faden & Beauchamp, 1986).

Informed consent from all participants will be obtained and participation will be purely voluntary, free of any coercion. This complies with the Belmont Report guidelines which stress on respect for persons, beneficence, and justice ( National Commission for the Protection of Human Subjects of Biomedical and Behavioral Research, 1978). Data will only be collected at two-time points, (1) prior to the exposure of students to problem-based learning (PBL) (pre-PBL) and after 8-months of exposure to PBL (post-PBL). The pre-post-test design will allow the researcher to assess the changes in students' critical thinking and communication skills over time ( Bryman, 2016).

The questionnaires will be administered through google forms to enhance behavioral consistency and reduce external factors. Using a controlled environment like this generates reliability and validity in how its administered ( Dillman *et al*., 2014). There will be a paper and pencil version for those who cannot fill out the google form. Utilizing paper-and-pencil questionnaires, enables potential accommodation for all participants, regardless of their ability to use digital applications. Having paper-based questionnaires will be particularly important, when conducting a research study wherein participant digital literacy levels are varied ( Sue & Ritter, 2007).

A mixed-mode approach, with paper questionnaires and the option of completing the questionnaires online via an encrypted site, will support participant preference and to protect their confidentiality. A pretest will also be undertaken to identify logistical problems and refine administration processes to ensure optimum validity and reliability. Questionnaires will be strategically given outside the high academic peak season to achieve the highest response and least participant burden.

### Timeline of data collection

Pre-PBL assessment: 15th October, 2024

Post-PBL assessment: 19th May, 2025.

### Data analysis method

Data analysis will be performed via IBM SPSS Statistics 26.0 or STATA version 13.0, which are widely used statistical software programs in educational research ( Field, 2024). Descriptive statistics will be applied to summarise participant demographic information and test scores. Measures of central tendency (mean, median, mode) and dispersion (standard deviation, range) will provide a general overview of the data distribution and help identify any outliers or anomalies ( Pallant, 2020).

Inferential tests, such as paired t tests or ANOVA, will be used to compare pre- and post-test scores. These are the best tests to apply when determining whether there are statistically significant differences between two time points in the measurement. Prior to applying the above tests, the normality of the data will be checked via tests such as the Shapiro Wilk test or the Kolmogorov Smirnov test. This ensures that the assumptions of the parametric tests are met ( Pallant, 2020).

Effect sizes will be calculated to determine the magnitude of the effects that PBL imposes on critical thinking and communication skills. Effect sizes provide a measure of practical significance, as well as the statistical significance of the findings ( Cohen, 2013). For example, Cohen's d or eta-square will be used to assess the strength of the relationship between the PBL and the measured outcomes. This is important for understanding the application of research in the outside world and for informing educational policy and practice ( Lakens, 2013).

To enhance internal validity and link observed effects to the PBL intervention, multiple regression analysis will be used to adjust for confounding variables such as prior academic performance, learning styles, and facilitator experience. Analysis of covariance (ANCOVA) adjusts for variation in baseline critical thinking and communication skills to minimize the effect of preintervention skill levels. Additionally, sensitivity analysis, including stratification and subgroup analyses, will assess the robustness of the findings across different confounder levels, ensuring credible causal inferences. Significance will be set at p < 0.05.

### Ethical considerations

Ethics are part and parcel of any research methodology and ensure that the research is conducted in a manner that is responsible, respectful, and carried out with integrity. It was in 1947 that the Nuremberg Code, and second, in 1964, the Helsinki Declaration laid the groundwork for modern-day research ethics, entrenching informed permission, volunteerism, protection from harm to persons, and protection of all individuals involved ( Experimentation, 1964; National Commission for the Protection of Human Subjects of Biomedical and Behavioral Research, 1978; Tribunal, 1949). The World Medical Association Declaration of Helsinki, 2013, echoes this when it mentions the following: "Respect for Persons: Respect for the individual, Beneficence, Non-Maleficence, and Justice" ( Association, 2013).

Contemporary researchers define what these are as the role of these ethics in research methods. Resnik (2018), for example, posits that ethical issues must be proposed in the design of the research, in the gathering of the data, and in the analysis. He stated that there are always ethical issues that may appear at any level of research and should be positively solved in advance. In turn, Shamoo and Resnik (2009) put much emphasis on the inevitability of such ethical conceptions in the scientific research process as respect for persons, beneficence, nonmaleficence, and justice.

In addition, the increase in the use of technology in research has also created other ethical issues. For example, the big data and artificial intelligence used in research stimulate challenging issues concerning privacy, confidentiality, and informed consent Mittelstadt *et al*. (2016). On the other hand, globalization has increased surface cultural sensitivity and inclusiveness both in the design and methodologies of studies.

Simply put, ethics in the research technique ensures that investigations are performed with integrity, respect, and accountability. A researcher needs to understand the tenets and norms under which ethics guide research and engage in solving emerging ethical issues in the process of research.

Therefore, this study will adhere strictly to ethical standards. Ethical approval for this study was obtained from the University for Development Studies Research and Ethics Review Board (Reference No. UDS/RB/0118/25) on May 5, 2025. The study will be conducted in full compliance with the ethical principles outlined in Declaration of Helsinki (2013). All participants will provide written informed consent before taking part in the study, and their anonymity and confidentiality will be maintained throughout.

### Informed consent

Informed consent is a critical ethical requirement in human research ( Beauchamp & Childress, 1994). In accordance with the Declaration of Helsinki of the World Medical Association (2013), the participants were given in-depth details regarding the study, such as its aim, procedures, risks, and benefits, to make ethical decisions regarding participation. In addition, a study conducted by Patel (2020) noted that written informed consent adds to transparency and guarantees compliance with ethical research practices. Hence, study participants will be provided with concise information on study aims and rights prior to signing informed consent documents.

### Confidentiality of data

The confidentiality of the participants is paramount in research ethics. According to GDPR (2016) and medical education research ethical guidelines, data safeguards should be put in place by researchers to ensure that participant data are not accessed without authorization. Anonymization of collected information is paramount in reducing the chances of breaches and participant privacy. Gadotti *et al*. (2024) assert that anonymization is also taken as a main method to make data made publicly available with minimal risks to privacy in research. El Emam *et al*. (2015) further stressed the need to deidentify sensitive data in educational and clinical research contexts to preserve confidentiality and meet ethical standards. All the data in this study were collected and stored in a manner that ensures anonymity, hence ensuring the confidentiality of the participants.

### Data management

Good data management practices are crucial for ensuring data integrity and security ( Bishop & Kuula-Luumi, 2017). The Wilkinson *et al*. (2016) FAIR guidelines (Findable, Accessible, Interoperable, and Reusable) present a way of managing research data ethically. Regular data backups need to be performed to prevent loss and data conservation ( Tenopir *et al*., 2020). In alignment with these best practices, in this research, deidentified information will be stored on a restricted-access secure server and ensures that the data are back-up regularly so that they are not lost.

## Discussion

This study is expected to investigate the effects of problem-based learning (PBL) on the learning of critical thinking, communication competencies, and satisfaction among first-year medical students at the University for Development Studies, School of Medicine (UDS-SoM). Drawing on constructivist, self-directed, and social learning philosophies, this study focuses on the active, collaborative, and student-centered premises that underlie PBL ( Bandura, 1977; Knowles, 1975; Piaget & Duckworth, 1970). The findings of this study are expected to make significant contributions to various domains.

The outcomes will direct the curriculum strategy in developing the first-year curriculum to better scaffold students into the PBL learning style. Transitional challenges, such as managing individual studies, group processes, and adjusting to a learner-centered model, require systematic orientation modules and clear learning expectations during initial introduction to PBL. The results can be used to inform curricular adjustments to the first-year curriculum to better assist new learners.

The study will also provide empirical data that can be utilized to develop faculty-centered evidence-based training workshops to facilitate PBL. Majority of PBL implementation problems are caused by differential tutor instruction, inadequate facilitator preparation, and inconsistency in group leadership as suggested by literature. The findings can be used to develop capacity-building programs to increase tutor effectiveness, communication, and feedback.

On the institutional front, this research offers evidence-based grounds for resource allocation towards improved classroom infrastructure, internet access, and increased facilitator‒student ratios. Policymaking regarding the optimal maximization of both student and facilitator support mechanisms in situations where there are constraining resources may be guided by data-informed insights from this work.

The findings from this study, may be useful for other medical schools in Ghana and across Sub-Saharan Africa seeking or developing PBL. Majority of such institutions have comparable limitations and would appreciate findings that are useful in their teaching and cultural contexts.

In summary, this study not only assesses the effect of PBL on early medical training but also aims to guide pedagogic practice, teacher education, and policy reform. The implementation of these findings in institution-level planning and national curriculum reform could have profound effects on enhancing the quality and equity of medical education in low-resource environments.

## Future applications

The findings from this study could serve as a catalyst for curriculum reform across other years of the medical program. Furthermore, lessons learned from this context can inform the development of PBL frameworks in similar resource-limited settings across Sub-Saharan Africa.

## Limitations

Despite the methodological strengths of a within-subjects design, several limitations must be acknowledged. First, all outcomes will be measured via self-report instruments, which may introduce social desirability bias and limit objectivity. Second, while students serve as their own controls, the 8-month interval between the pre- and post-test assessments may be influenced by other intervening variables, including exposure to other learning activities or external academic influences. Third, although the same trained facilitators will be used throughout the intervention to ensure standardization, variations in facilitation style and student group dynamics may still influence outcomes.

Moreover, the lack of an external control group constrains the ability to isolate PBL as the sole factor responsible for observed changes. Finally, the single-institution scope of the study limits the generalizability of the results to other academic or geographical contexts. Future studies incorporating longitudinal tracking and mixed-methods evaluation may offer more comprehensive insights into the long-term effect and causal pathways of PBL on student competencies.

## Dissemination

The study findings will be disseminated through presentations at academic board meetings and curriculum review workshops held at the UDS. The results will also be submitted for publication in peer-reviewed journals and at national and international medical education conferences.

## Declarations

### Ethics approval and consent to participate

Ethical approval for the study was obtained from the University for Development Studies, School of Medicine Research and Ethics Committee (Reference No. **UDS/RB/0118/25**). All participants will provide written informed consent before participation.

## Consent for publication

Not applicable.

## Data Availability

No data are associated with this article.
