# Effects of Problem-Based Learning on Critical Thinking, Communication Skills, and Satisfaction Among First-Year Medical Students in Ghana: A study protocol

**DOI:** 10.12688/mep.21103.2

**Published:** 2025-09-10

**Authors:** Bruce Ayabilla Abugri, Maxwell Ateni Assibi, Anthony Amalba, Patience Kanyiri Gaa, Sophia E. A. Kpebu, Victor Mogre

**Affiliations:** 1 Department of Health Professions Education and Innovative Learning, School of Medicine Professions Education and Innovative Learning, University for Development Studies, Tamale, Northern Region, 00233, Ghana; 2 Department of Pharmaceutical Practice, School of Pharmacy and Pharmaceutical Sciences, University for Development Studies, Tamale, Northern Region, 00233, Ghana; 3 Department of Dietetics, School of Allied Health Science, University for Development Studies, Tamale, Northern Region, 00233, Ghana

**Keywords:** Problem-based learning, critical thinking, communication skills, student satisfaction, medical education, Ghana

## Abstract

**Background:**

Problem-Based Learning (PBL) is increasingly used in medical education to develop critical thinking, communication, and learner autonomy. Although widely studied in developed countries, evidence from low-resource settings like Ghana, particularly involving first-year students, remains limited. This study seeks to address this gap by assessing the impact of PBL on critical thinking, communication skills, and satisfaction levels among novice medical students.

**Methods:**

This longitudinal pre-test-post-test study will be conducted at the University for Development Studies, School of Medicine. All first-year students who meet the eligibility criteria will be enrolled. Participants will undergo baseline (pre-PBL) and follow-up (post-PBL) assessments using the Critical Thinking Questionnaire (licensed under a Creative Commons Attribution 4.0 International License (CC BY 4.0) and Interpersonal Communication Competency Skills Scale (ICCS). A 5-point Likert scale will be used to measure student satisfaction after eight months of exposure to PBL. Statistical analyses including paired t-tests, ANOVA, and regression will be performed using SPSS v26.0. to assess changes through the mean scores.

**Discussion:**

The study will provide context-specific insights on the effectiveness of PBL in enhancing key competencies among first-year medical students. Findings will inform curriculum development, tutor training, and educational policy in Ghana and similar settings.

**Clinical trial registration number:**

Not applicable.

## Introduction

### Background

Medical education has evolved from apprenticeship-style training to structured, competency-based approaches that emphasize lifelong learning. The Flexner Report of 1910 established a scientifically rigorous model that shaped curricula worldwide ( Cooke *et al.*, 2010; Frenk *et al.*, 2010) . More recently, competency-based education, simulation, and student-centred learning have been introduced to better prepare learners for the complex demands of modern practice ( Ellaway & Masters, 2008; Frank *et al.*, 2010; Harden, 2020; Schuwirth & van der Vleuten, 2020).

Problem-Based Learning (PBL), first introduced at McMaster University in the late 1960s ( Neufeld & Barrows, 1974), is a student-centred pedagogy that uses authentic problems as the basis for learning ( Barrows, 1986). By shifting the focus from teacher-led instruction to collaborative inquiry, PBL promotes self-directed learning, critical reasoning, and teamwork ( Dolmans *et al.*, 2016; Neville, 2009).

Evidence indicates that PBL enhances a range of competencies essential for medical education, including critical thinking, communication, problem solving, and independent learning ( Dolmans *et al.*, 2016; Neville, 2009; Vargas-Rodríguez *et al.*, 2021). Through active engagement with complex case scenarios, students develop skills that prepare them for both academic progression and professional practice.

Despite its advantages, PBL is sometimes criticized as resource intensive and challenging for novice learners, particularly first-year students who may lack the metacognitive skills to thrive in self-directed environments ( Manuaba *et al.*, 2022). Concerns have also been raised regarding facilitator variability and potential overlap with traditional curricula. Nevertheless, most studies highlight that the benefits outweigh these limitations.

Systematic reviews support the effectiveness of PBL in undergraduate medical education. A scoping review by Trullàs *et al.* (2022) concluded that PBL not only facilitates knowledge acquisition but also helps students develop transferable competencies relevant to clinical practice. Similarly, a meta-analysis by Sayyah *et al.* (2017) found consistent positive effects of PBL on academic achievement.

In Ghana, PBL was introduced in Ghana especially at the University for Development Studies (UDS) School of Medicine and the University of Cape Coast in 2007 ( Amoako-Sakyi & Amonoo-Kuofi, 2015; Mogre & Amalba, 2015). The UDS implementation represents a significant educational innovation in a resource-limited setting, where evidence on the outcomes of PBL remains limited.

Although PBL is respected for fostering analytical reasoning, problem solving, and interpersonal communication, its effectiveness for first-year medical students remains unclear. Studies suggest that transition difficulties including coping with independent study, group dynamics, and professional identity formation may shape outcomes ( Ocheretnyuk *et al.*, 2018; Picton *et al.*, 2022). Manuaba *et al.* (2022) further argue that contextual and implementation factors strongly influence PBL’s impact. These observations highlight the need for context-specific research on first-year medical students, particularly in low-resource environments.

This study draws on constructivist learning theory ( Piaget & Duckworth, 1970; Vygotsky, 1978), self-directed learning theory ( Knowles, 1975), and social learning theory ( Bandura, 1977). Together, these frameworks explain how PBL fosters active knowledge construction, learner autonomy, and collaborative inquiry. The conceptual model positions PBL as the independent variable influencing critical thinking and communication, mediated by processes such as metacognition, social interaction, and cognitive engagement, while accounting for confounding factors including prior academic preparation and facilitator competence.

In summary, although PBL is widely implemented, little is known about its effects on first-year medical students in low-resource African settings. To address this gap, the present study protocol outlines a longitudinal pre–posttest design to evaluate the impact of PBL on critical thinking, interpersonal communication competence, and student satisfaction among first-year medical students at the UDS School of Medicine.

### Problem statement

Problem-based learning (PBL) is increasingly promoted in health professional education because of its potential to improve the critical thinking, communication, and group working skills needed in clinical judgement and professional practice ( Dolmans *et al*., 2016; Neville, 2009). Medical education has experienced an immense paradigm shift to student-centered instruction since PBL was introduced.

However, issues arise regarding its specific effectiveness among first-year medical students who are just being introduced to self-directed and collaborative learning environments. A scoping review was conducted to examine the literature concerning the effects of PBL on critical thinking and communication skills in first-year undergraduate medical students in high- and low-resource settings. Although the review confirmed the potential of PBL to enhance these skills, two significant gaps were identified. The first was a contextual gap: no African countries, such as Ghana, where education and health systems may be significantly different from high-resource contexts. The second was a conceptual gap: there was only one study ( Yadav *et al*., 2018) in Nepal that focused on first-year medical students. This study measured generic skills such as analytical thinking, collaborative practice, and active listening via a cross-sectional design and self-report questionnaire, precluding its depth and capacity to infer causality. These findings reinforce the need for context specific, methodologically robust research on the effects of PBL among first-year medical students in resource-constrained settings, critical thinking and communication skills development and student satisfaction.

First-year students commonly find it difficult to cope with PBL, including handling independent study, dealing with group work, and actively participating in peer discussion ( Manuaba *et al*., 2022). Such transition challenges are also exacerbated when there is poor faculty direction, poor physical facilities, and inadequate formal training in communication skills ( Mughal & Shaikh, 2018). Student satisfaction, which is highly related to engagement, motivation, and learning achievement, has also been inappropriately balanced in the literature. Exceedingly high levels of satisfaction with PBL's interactive and collaborative structure have been cited in some papers ( AlHaqwi *et al*., 2015; Errabti *et al*., 2024), whereas others voice alarms over time, alignment in assessment, and facilitator preparation ( Animaw & Asaminew, 2023; Barnawi *et al*., 2024; Mohammed *et al*., 2024).

These gaps, particularly in relation to first-year studies in various African contexts, raise important concerns regarding the pedagogical integrity and contextual relevance of PBL. Longitudinal research is crucial to better understand novice learners' engagement with PBL and how this method affects long-term critical thinking skills, communication abilities, and levels of satisfaction. The findings will form the basis of curricular refinements and help inform implementational practices across similarly constrained academic environments.

### Objectives and research questions

***General objective***

To determine the effects of PBL on critical thinking, communication skills, and satisfaction levels among first-year medical students.

***Specific objectives***

1. To assess changes in students’ critical thinking skills after exposure to PBL.
2. To examine the effect of PBL on students’ communication skills.
3. To assess students’ satisfaction with PBL-based instruction.

***Research questions***

1. What is the effect of PBL on critical thinking skills among first-year medical students at the UDS?
2. What is the effect of PBL on communication skills?
3. What is the level of student satisfaction with their experience with PBL?

### Significance of the study

This study provides essential empirical evidence on how problem-based learning (PBL) influences critical thinking, communication skills, and satisfaction levels among first-year medical students in a low-resource context. While PBL has been integrated into medical curricula worldwide, existing research has not adequately explored its effect at the foundational level of training. This gap is especially visible in two areas: contextual and knowledge-based.

The contextual gap refers to the absence of studies conducted in African settings, particularly Ghana, where the educational, cultural, and infrastructural realities differ significantly from those in which most PBL research has been conducted. A recent scoping review by the research team revealed that all included studies on the effects of PBL were based in Asia, with no representation from African countries. This spatial disparity limits the generalizability of current findings to universities such as the University for Development Studies (UDS), which are plagued by problems such as larger class sizes, limited access to online resources, and fewer trained facilitators ( Jdaitawi, 2020; Mughal & Shaikh, 2018). The UDS, as one of the pioneers of Ghanaian universities to implement PBL ( Amoako-Sakyi & Amonoo-Kuofi, 2015; Mogre & Amalba, 2015), presents a suitable context in which to investigate how the approach performs in real-world, resource-limited environments.

The knowledge deficit represents the lack of evidence regarding the effect of PBL among first-year medical students in terms of critical thinking and communication skills development and students’ satisfaction. While various studies have revealed improved critical thinking and problem-solving among medical students taught via PBL ( Asad *et al*., 2015; Pu *et al*., 2019), virtually all of these studies involve upper-level students or combined samples that are not conducive to the first-year learner effect.

Only one study in the reviewed literature focused exclusively on first-year medical students and employed a descriptive, cross-sectional approach based solely on self-reported perceptions, making it difficult to establish any measurable change over time ( Yadav *et al*., 2018).

This study addresses both gaps through a quantitative pre-test‒post-test design that measures self-perceived changes in critical thinking and communication skills, as well as levels of satisfaction following PBL exposure. Unlike previous studies that relied on single-point feedback evidence, this research applies validated instruments to capture changes over time. Measuring outcomes in this way provides a more objective assessment of PBL’s effect on key educational competencies.

Critical thinking is a fundamental skill in medical education that influences diagnostic reasoning, clinical decision-making, and lifelong learning ( Ennis, 2018; Facione, 2011). PBL is widely believed to support its development by engaging students in inquiry, reflection, and peer interaction ( Dolmans *et al*., 2016; Neville, 2009). However, little is known about how effectively PBL fosters critical thinking in first-year students who may not yet have the foundational scientific knowledge or the metacognitive skills necessary to fully benefit from self-directed learning.

Similarly, communication skills are essential. Good and respectful interaction with patients, colleagues, and healthcare teams depends on a student’s ability to listen, express ideas clearly, and collaborate within interdisciplinary settings ( Kurtz *et al*., 2017; Silverman *et al*., 2016). PBL introduces students to group discussions, case presentations, and role-based learning, all of which can enhance communication competence. However, most available studies focus on later stages of training or are qualitative in nature ( Hande *et al*., 2015), offering limited insight into the early development of communication skills through PBL among new medical students.

Learner satisfaction also plays a key role in academic performance, motivation, and course engagement. Medical students’ perceptions of PBL vary widely, and dissatisfaction may stem from workload demands, a lack of clarity in learning outcomes, or weak tutor facilitation ( AlHaqwi *et al*., 2015; Errabti *et al*., 2024; Nahar *et al*., 2014). Understanding satisfaction levels through quantitative analysis provides measurable data that can inform improvements in PBL delivery and tutor training, particularly at the introductory level of medical education.

At the institutional level, the findings will enable the School of Medicine at the UDS to evaluate how well PBL is serving its intended goals. This includes examining whether students are acquiring the competencies the curriculum is designed to promote and whether they feel supported in the learning process. The evidence from this study will help guide decisions about resource allocation, facilitator development, and potential adjustments in how PBL is structured or introduced during the first year.

Other medical schools in Ghana and similar resource-limited contexts may also benefit from the findings. Many of these institutions are in the early stages of adopting PBL and often lack local data to inform implementation strategies. The use of context-relevant evidence from UDSs can provide a reference point for best practices or identify areas requiring institutional adaptation.

This research also contributes to broader policy discussions within medical education reform. Competency-based education models, which are increasingly endorsed at the regional and global levels, emphasize measurable outcomes and skill development over content delivery alone ( Frank *et al*., 2010). PBL is frequently presented as a vehicle for this transition. However, its effectiveness must be validated across diverse contexts through systematic, outcome-based research. Findings from this study will help build that evidence base, offering a clearer picture of how PBL performs in underrepresented regions such as sub-Saharan Africa.

In summary, this study is pertinent both in its setting and methodological contribution. This study provides measurable data on the effects of PBL on critical thinking, communication skills, and student satisfaction among first-year medical students at the UDS. This study addresses obvious gaps in the literature, strengthens evidence-based curriculum innovation, and contributes to the local and regional understanding of PBL's contribution to medical education. The results could enhance teaching and learning not only at the UDS but also at other universities experiencing the same learning and resource constraints.

## Methodology

### Study design

This research uses a quantitative longitudinal pretest and post-test design to evaluate the effect of problem-based learning (PBL) on critical thinking, communication skills, and satisfaction among first-year medical students at the University for Development Studies, School of Medicine (UDS-SoM), Tamale. The design allows for measurement of change at the individual level over time and is appropriate for the evaluation of the causal effect of education interventions via standard instruments ( Creswell & Creswell, 2017).

### Study setting

The study will be conducted at the UDS-SoM, which offers a PBL curriculum. The school is affiliated with the Tamale Teaching Hospital for the clinical training.

### Participants and inclusion criteria

***Target population***

All first-year medical students enrolled in the 2024–2025 academic year at UDS-SoM.

They are selected because they are fresh from their premedical school training and are not yet to exposed to PBL in this institutional context.

***Inclusion criteria***

Enrolled as first-year medical students at the UDS-SoM during the study period

The participants will be first-year medical students aged between 18 and above.

Voluntary participation and willingness to provide informed consent

No prior exposure to PBL at any learning institution

In student screening for prior experience with PBL, a screening questionnaire will be given during the process of providing consent. The identified students will be excluded for the purpose of maintaining internal validity and for ensuring that the study assesses the effect of the UDS's implementation of PBL specifically.

### Sampling strategy and sample size

A census sampling approach will be adopted, inviting all potentially eligible first-year students to participate. This approach reduces selection bias and offers maximum representation of the cohort ( Etikan *et al*., 2016). To justify the adequacy of the sample, a priori power analysis was conducted using G*Power 3.1 for a two-tailed paired t test with an alpha level of .05 and statistical power of 0.80. The analysis indicated that a minimum of 34 participants will be required to detect a medium effect size (d = 0.50), 51 participants to detect a smaller effect (d = 0.40), and 90 participants to detect a small-to-moderate effect ( *d* = 0.30). Given an expected class size of approximately 110 students, the study is anticipated to achieve a sample size that exceeds the minimum requirements, thereby ensuring sufficient power to detect meaningful within-group differences across the planned outcomes ( Cohen, 2013).

### Intervention

Students will receive two times weekly, PBL sessions guided by facilitator for eight-month. Each session is based on case scenarios prepared from basic medical sciences that form the foundation of medicine. Students define learning objectives, learn independently, and return to share and discuss outcomes in groups. Facilitators foster questioning and group collaboration without providing direct solutions, encouraging autonomy and collaboration.

### Implementation fidelity and tutor training

The University for Development Studies (UDS) School of Medicine has adopted Problem-Based Learning (PBL) as its primary pedagogical approach since 2007 ( Mogre & Amalba, 2015). Accordingly, the present study did not introduce PBL but rather evaluated its effect on the development of critical thinking skills, interpersonal communication competencies, and student satisfaction among first-year medical students as they progressed through PBL sessions across trimesters in their first academic year.

Implementation fidelity was safeguarded through established institutional mechanisms. The Department of Health Professions Education and Innovative Learning (DHPEIL) conducts annual capacity-building and staff development workshops for all PBL tutors. These workshops provide training in the philosophy of PBL, small-group facilitation, independent study guidance, group discussions, task preparation, presentation skills, assessment strategies, and group process management. By institutionalizing such structured training annually, UDS ensures consistency in PBL delivery and sustains alignment with international standards in health professions education.

Therefore, students in the 2024/2025 academic year will experience PBL within a natural and standardized institutional framework facilitated by trained tutors. The current investigation intends to focus on only assessing the outcomes of PBL specifically critical thinking skills, interpersonal communication competency skills, and students’ satisfaction using a longitudinal pre-posttest design.

Ongoing monitoring of PBL at UDS Schoof Medicine are as follows:

1. Peer observations of PBL tutorials using standardized checklists.
2. Student feedback surveys after each trimester.
3. Refresher sessions to address identified gaps and ensure consistency across facilitators.

### Data collection instruments

The study will utilize three standard instruments: the Critical Thinking Questionnaire (CThQ), the Interpersonal Communication Competency Scale (ICCS) and a 5-point Likert scale. The CThQ, licensed under a Creative Commons Attribution 4.0 International License (CC BY 4.0) will be used to measure critical thinking skills, the interpersonal communication competency scale will measure interpersonal communication competency skills, and the 5-point Likert scale will measure students' satisfaction. All the instruments have been used in previous research ( Errabti *et al.*, 2024; Ross *et al.*, 2014; Younis, 2025) and are widely used in education studies ( Kobylarek *et al*., 2022; Rubin & Martin, 1994).

The CThQ is a reliable and valid tool for assessing critical thinking skills, with a Cronbach's alpha over 0.84 indicating that the tool is reliable enough. The questionnaire possesses a number of items for assessing critical thinking in various dimensions, such as remembering, understanding, applying, analysing, evaluating, and creating. The ICCS is a 30-item measure consisting of five subscales of interpersonal communication competence: self-disclosure, empathy, social relaxation, assertiveness, and altercentrism. A pilot study was utilized in developing the reliability of the scales, and a Cronbach's alpha of 0.84 and above was deemed acceptable reliability. Face and content validity will be determined by expert and participant ratings to determine whether the instruments are suitable for the task. The scales are piloted repeatedly to ensure that they are internally consistent and capable of measuring the constructs under investigation.

### Data collection procedure

Once ethical approval is acquired from the University for Development Studies Ethical Review Board, permission to proceed with the study will be sought from the University for Development Studies School of Medicine authorities. This is necessary to ensure that the study is in line with institutional policy and ethical practice ( Creswell & Creswell, 2017). Information sessions will be organized to inform the students about the purpose of the study, the procedures followed, and the rights of the participants. Such sessions are required to ensure transparency and to acquire informed consent, which is the basis of ethical research ( Faden & Beauchamp, 1986).

Informed consent from all participants will be obtained and participation will be purely voluntary, free of any coercion. This complies with the Belmont Report guidelines which stress on respect for persons, beneficence, and justice ( National Commission for the Protection of Human Subjects of Biomedical and Behavioral Research, 1978). Data will only be collected at two-time points, (1) prior to the exposure of students to problem-based learning (PBL) (pre-PBL) and after 8-months of exposure to PBL (post-PBL). The pre-post-test design will allow the researcher to assess the changes in students' critical thinking and communication skills over time ( Bryman, 2016).

The questionnaires will be administered through google forms to enhance behavioral consistency and reduce external factors. Using a controlled environment like this generates reliability and validity in how its administered ( Dillman *et al*., 2014). There will be a paper and pencil version for those who cannot fill out the google form. Utilizing paper-and-pencil questionnaires, enables potential accommodation for all participants, regardless of their ability to use digital applications. Having paper-based questionnaires will be particularly important, when conducting a research study wherein participant digital literacy levels are varied ( Sue & Ritter, 2007).

A mixed-mode approach, with paper questionnaires and the option of completing the questionnaires online via an encrypted site, will support participant preference and to protect their confidentiality. A pretest will also be undertaken to identify logistical problems and refine administration processes to ensure optimum validity and reliability. Questionnaires will be strategically given outside the high academic peak season to achieve the highest response and least participant burden.

### Timeline of data collection

Pre-PBL assessment: 15th October, 2024

Post-PBL assessment: 19th May, 2025.

### Data analysis method

Data analysis will be performed via IBM SPSS Statistics 26.0 or STATA version 13.0, which are widely used statistical software programs in educational research ( Field, 2024). Descriptive statistics will be applied to summarise participant demographic information and test scores. Measures of central tendency (mean, median, mode) and dispersion (standard deviation, range) will provide a general overview of the data distribution and help identify any outliers or anomalies ( Pallant, 2020).

Inferential tests, such as paired t tests or ANOVA, will be used to compare pre- and post-test scores. These are the best tests to apply when determining whether there are statistically significant differences between two time points in the measurement. Prior to applying the above tests, the normality of the data will be checked via tests such as the Shapiro Wilk test or the Kolmogorov Smirnov test. This ensures that the assumptions of the parametric tests are met ( Pallant, 2020).

Effect sizes will be calculated to determine the magnitude of the effects that PBL imposes on critical thinking and communication skills. Effect sizes provide a measure of practical significance, as well as the statistical significance of the findings ( Cohen, 2013). For example, Cohen's d or eta-square will be used to assess the strength of the relationship between the PBL and the measured outcomes. This is important for understanding the application of research in the outside world and for informing educational policy and practice ( Lakens, 2013).

To enhance internal validity and link observed effects to the PBL intervention, multiple regression analysis will be used to adjust for confounding variables such as prior academic performance, learning styles, and facilitator experience. Analysis of covariance (ANCOVA) adjusts for variation in baseline critical thinking and communication skills to minimize the effect of preintervention skill levels. Additionally, sensitivity analysis, including stratification and subgroup analyses, will assess the robustness of the findings across different confounder levels, ensuring credible causal inferences. Significance will be set at p < 0.05.

### Ethical considerations

Ethics are part and parcel of any research methodology and ensure that the research is conducted in a manner that is responsible, respectful, and carried out with integrity. It was in 1947 that the Nuremberg Code, and second, in 1964, the Helsinki Declaration laid the groundwork for modern-day research ethics, entrenching informed permission, volunteerism, protection from harm to persons, and protection of all individuals involved ( Experimentation, 1964; National Commission for the Protection of Human Subjects of Biomedical and Behavioral Research, 1978; Tribunal, 1949). The World Medical Association Declaration of Helsinki, 2013, echoes this when it mentions the following: "Respect for Persons: Respect for the individual, Beneficence, Non-Maleficence, and Justice" ( Association, 2013).

Contemporary researchers define what these are as the role of these ethics in research methods. Resnik (2018), for example, posits that ethical issues must be proposed in the design of the research, in the gathering of the data, and in the analysis. He stated that there are always ethical issues that may appear at any level of research and should be positively solved in advance. In turn, Shamoo and Resnik (2009) put much emphasis on the inevitability of such ethical conceptions in the scientific research process as respect for persons, beneficence, nonmaleficence, and justice.

In addition, the increase in the use of technology in research has also created other ethical issues. For example, the big data and artificial intelligence used in research stimulate challenging issues concerning privacy, confidentiality, and informed consent Mittelstadt *et al*. (2016). On the other hand, globalization has increased surface cultural sensitivity and inclusiveness both in the design and methodologies of studies.

Simply put, ethics in the research technique ensures that investigations are performed with integrity, respect, and accountability. A researcher needs to understand the tenets and norms under which ethics guide research and engage in solving emerging ethical issues in the process of research.

Therefore, this study will adhere strictly to ethical standards. Ethical approval for this study was obtained from the University for Development Studies Research and Ethics Review Board (Reference No. UDS/RB/0118/25) on May 5, 2025. The study will be conducted in full compliance with the ethical principles outlined in Declaration of Helsinki (2013). All participants will provide written informed consent before taking part in the study, and their anonymity and confidentiality will be maintained throughout.

### Informed consent

Informed consent is a critical ethical requirement in human research ( Beauchamp & Childress, 1994). In accordance with the Declaration of Helsinki of the World Medical Association (2013), the participants will be given in-depth details regarding the study, such as its aim, procedures, risks, and benefits, to make ethical decisions regarding participation. In addition, a study conducted by Patel (2020) noted that written informed consent adds to transparency and guarantees compliance with ethical research practices. Hence, study participants will be provided with concise information on study aims and rights prior to signing informed consent documents.

### Confidentiality of data

The confidentiality of the participants is paramount in research ethics. According to GDPR (2016) and medical education research ethical guidelines, data safeguards should be put in place by researchers to ensure that participant data are not accessed without authorization. Anonymization of collected information is paramount in reducing the chances of breaches and participant privacy. Gadotti *et al*. (2024) assert that anonymization is also taken as a main method to make data made publicly available with minimal risks to privacy in research. El Emam *et al*. (2015) further stressed the need to deidentify sensitive data in educational and clinical research contexts to preserve confidentiality and meet ethical standards. All the data in this study will be collected and stored in a manner that ensures anonymity, hence ensuring the confidentiality of the participants.

### Data management

Good data management practices are crucial for ensuring data integrity and security ( Bishop & Kuula-Luumi, 2017). The Wilkinson *et al*. (2016) FAIR guidelines (Findable, Accessible, Interoperable, and Reusable) present a way of managing research data ethically. Regular data backups need to be performed to prevent loss and data conservation ( Tenopir *et al*., 2020). In alignment with these best practices, in this research, deidentified information will be stored on a restricted-access secure server and ensures that the data are back-up regularly so that they are not lost.

## Discussion

This study is expected to investigate the effects of problem-based learning (PBL) on the learning of critical thinking, interpersonal communication competencies, and satisfaction among first-year medical students at the University for Development Studies, School of Medicine (UDS-SoM). Drawing on constructivist, self-directed, and social learning philosophies, this study focuses on the active, collaborative, and student-centered premises that underlie PBL ( Bandura, 1977; Knowles, 1975; Piaget & Duckworth, 1970). The findings of this study are expected to make significant contributions to various domains.

The outcomes will direct the curriculum strategy in developing the first-year curriculum to better scaffold students into the PBL learning style. Transitional challenges, such as managing individual studies, group processes, and adjusting to a learner-centered model, require systematic orientation modules and clear learning expectations during initial introduction to PBL. The results can be used to inform curricular adjustments to the first-year curriculum to better assist new learners.

The study will also provide empirical data that can be utilized to develop faculty-centered evidence-based training workshops to facilitate PBL. Majority of PBL implementation problems are caused by differential tutor instruction, inadequate facilitator preparation, and inconsistency in group leadership as suggested by literature. The findings can be used to develop capacity-building programs to increase tutor effectiveness, communication, and feedback.

On the institutional front, this research will offer evidence-based grounds for resource allocation towards improved classroom infrastructure, internet access, and increased facilitator‒student ratios. Policymaking regarding the optimal maximization of both student and facilitator support mechanisms in situations where there are constraining resources may be guided by data-informed insights from this work.

The findings from this study, will be useful for other medical schools in Ghana and across Sub-Saharan Africa seeking or developing PBL. Majority of such institutions may have comparable limitations and will appreciate findings that are useful in their teaching and cultural contexts.

In summary, this study will not only assess the effect of PBL on early medical training but also guide pedagogic practice, teacher education, and policy reform. The implementation of these findings in institution-level planning and national curriculum reform could have profound effects on enhancing the quality and equity of medical education in low-resource environments.

## Future applications

The findings from this study will serve as a catalyst for curriculum reform across other years of the medical program. Furthermore, lessons that will be learned from this context can inform the development of PBL frameworks in similar resource-limited settings across Sub-Saharan Africa.

## Limitations

Despite the methodological strengths of employing a longitudinal within-subjects design, several limitations must be acknowledged in this protocol. First, all outcomes will be measured using self-report instruments, which may introduce social desirability bias and limit objectivity. Second, although students will serve as their own controls, the eight-month interval between pre- and post-test assessments may expose participants to other learning experiences or external academic influences that could affect the results. Third, while the use of trained facilitators and annual capacity-building workshops will help maintain standardization, differences in facilitation style and group dynamics may still influence outcomes.

In addition, the absence of an external control group constrains the ability to attribute observed changes solely to PBL. Although a census approach will invite all 110 eligible first-year students, the relatively small cohort size in a single institution may limit the generalizability of the findings to other contexts, particularly within diverse low-resource settings.

## Dissemination

The findings of this study will be disseminated through presentations at annual academic board meetings and curriculum review workshops at the University for Development Studies. In addition, the results will be submitted for publication in peer-reviewed journals and presented at national and international medical education conferences

## Declarations

### Ethics approval and consent to participate

Ethical approval for the study was obtained from the University for Development Studies, School of Medicine Research and Ethics Committee (Reference No. **UDS/RB/0118/25**). All participants will provide written informed consent before participation.

## Consent for publication

Not applicable.

## Data Availability

No data are associated with this article.
