# Supplementary material for: Effects of Problem-Based Learning on Critical Thinking, Communication Skills, and Satisfaction Among First-Year Medical Students in Ghana: A study protocol
Source: MedEdPublish (2016). 2025 Sep 10;15:31. Originally published 2025 Jul 9. [Version 2] doi: 10.12688/mep.21103.2 (PMC12489453; doi:10.12688/mep.21103.2)
Supplement: Peer review history

# Reviewer report: Reviewer response for version 2

**DOI:** 10.21956/mep.22779.r43538

**Published:** 2025-10-01

**Authors:** Asmaa Abdel Nasser

**Affiliations:** 1 Suez Canal University, Ismailia, Ismailia Governorate, Egypt

This is a well-written and relevant study protocol addressing an important gap in the literature—namely, the impact of Problem-Based Learning (PBL) in low-resource settings and among first-year medical students. The authors clearly articulate the rationale, objectives, and significance of their work. The protocol has been considerably strengthened in its revised version, particularly with improved clarity, methodological transparency, and alignment with protocol standards.

 Strengths

1. Relevance and originality
  - The study addresses an underexplored area: the effect of PBL in Ghana and similar low-resource contexts.
  - The focus on first-year medical students is highly pertinent, as most prior PBL research targets later years.
2. Theoretical foundation
  - The manuscript grounds its design in constructivist, self-directed, and social learning theories, enhancing conceptual rigor.
3. Methodological clarity
  - The use of a longitudinal pre–post design is appropriate to measure change over time.
  - Sample size justification with power analysis is included.
  - Instruments (CThQ, ICCS, Likert satisfaction scale) are validated and relevant.
4. Implementation fidelity
  - Annual tutor training and monitoring mechanisms strengthen internal validity and ensure standardized PBL delivery.
5. Transparency
  - Ethical approval is documented, and limitations are openly acknowledged.

Areas for Improvement

1. Literature Review
  - While the introduction has been streamlined, some references are still overly concentrated on global perspectives. A deeper comparison with existing African or LMIC PBL literature could further situate the study.
2. Outcome Measures
  - Although validated, all instruments are self-report. The authors might consider including at least one performance-based assessment (e.g., observed group work or OSCE-related communication station) to triangulate outcomes.
3. Satisfaction Assessment
  - The 5-point Likert scale is mentioned but not described in detail. Clarifying whether it is adapted from prior studies or newly developed would improve replicability.
4. Potential Confounders
  - While regression is planned, the manuscript could explicitly list potential confounders (e.g., gender, prior academic performance, socio-economic background) and how they will be measured.
5. Dissemination Plan
  - The dissemination section could be expanded to include how findings will be fed back to students and faculty, beyond academic boards and conferences.

Minor Points

- Some typographical and grammatical inconsistencies remain (e.g., “students are not yet to exposed” → should be “not yet exposed”).
- The timeline (October 2024 to May 2025) is well defined, but data analysis dates and dissemination phases could be more explicitly linked to milestones.
- The Limitations section could highlight the possibility of attrition bias given the long follow-up period.

Recommendation

 Approved with minor reservations.

 This study protocol is methodologically sound and highly relevant for advancing competency-based medical education in resource-limited contexts. Addressing the minor points above would further enhance clarity, replicability, and scholarly contribution.

Is the study design appropriate for the research question?

Yes

Is the rationale for, and objectives of, the study clearly described?

Yes

Are sufficient details of the methods provided to allow replication by others?

Yes

Are the datasets clearly presented in a useable and accessible format?

Yes

Reviewer Expertise:

Medical Education

I confirm that I have read this submission and believe that I have an appropriate level of expertise to confirm that it is of an acceptable scientific standard, however I have significant reservations, as outlined above.

# Reviewer report: Reviewer response for version 2

**DOI:** 10.21956/mep.22779.r43449

**Published:** 2025-09-27

**Authors:** Nora Ramos-Vallecillo

**Affiliations:** 1 Universidad de Zaragoza, Zaragoza, Spain

Thank you for reflecting all the proposed changes and taking into account the comments made. I believe that the article has gained substantially in terms of readability and replicability.

Is the study design appropriate for the research question?

Yes

Is the rationale for, and objectives of, the study clearly described?

Yes

Are sufficient details of the methods provided to allow replication by others?

Partly

Are the datasets clearly presented in a useable and accessible format?

No

Reviewer Expertise:

NA

I confirm that I have read this submission and believe that I have an appropriate level of expertise to confirm that it is of an acceptable scientific standard.

# Reviewer report: Reviewer response for version 1

**DOI:** 10.21956/mep.22606.r42627

**Published:** 2025-08-30

**Authors:** Nora Ramos-Vallecillo

**Affiliations:** 1 Universidad de Zaragoza, Zaragoza, Spain

**1. Is the work clearly and precisely presented, and does it cite current literature?**

 The manuscript addresses a relevant and timely topic, particularly in the current educational context: the applicability of active teaching methodologies—widely validated in European and American university settings—to economically disadvantaged environments. The topic is pertinent and of interest. However, the presentation of the manuscript would benefit from greater clarity and coherence in both writing and structure. For example, the introduction includes content that would be more appropriately placed in the discussion section. Although references are included, the theoretical framework could be strengthened by incorporating more recent and specific literature on similar studies conducted in low-resource settings.

**2. Is the study design appropriate and is the work technically sound?**

 The proposed design is interesting, but the study has not been carried out. It is a theoretical research proposal, which significantly limits its empirical value and its contribution to scientific knowledge. The argumentation is solid, but the lack of actual implementation weakens the impact of the article. Readers in the scientific community generally expect access to concrete data derived from completed research.

**3. Are sufficient details of methods and analysis provided to allow replication by others?**

 The methodology is described in a general and structured manner, but it lacks sufficient operational details to allow replication by other researchers. It would be necessary to include more specific information regarding variables, instruments, procedures, and contextual conditions to ensure the replicability of the proposed study in future empirical work.

**4. If applicable, is the statistical analysis and its interpretation appropriate?**

 The article does not include any statistical analysis or data interpretation, as it is solely a research proposal. This absence prevents any assessment of the validity of the results or the relevance of the conclusions.

**5. Are the limitations of the research acknowledged?**

 The limitations of the study are not explicitly discussed by the authors, although they are evident. The primary limitation is that the study remains a theoretical proposal and has not been implemented, meaning there are no actual data or results. Moreover, if it were to be conducted, the proposed sample size—approximately 100 students—would be relatively small for drawing generalizable conclusions, especially considering potential contextual variables (socio-cultural, institutional, etc.).

**6. Are all underlying data to support the results available to ensure full reproducibility?**

 No empirical data are presented in the manuscript, so there is no information available to verify results or ensure the reproducibility of the study.

**7. Are the conclusions adequately supported by the results?**

 Any form of empirical analysis does not support the conclusions, as the study has not been conducted. Therefore, the final statements are speculative in nature, which significantly reduces the scientific strength of the manuscript.

Is the study design appropriate for the research question?

Yes

Is the rationale for, and objectives of, the study clearly described?

Yes

Are sufficient details of the methods provided to allow replication by others?

Partly

Are the datasets clearly presented in a useable and accessible format?

No

Reviewer Expertise:

Active learning methodologies in higher education

I confirm that I have read this submission and believe that I have an appropriate level of expertise to state that I do not consider it to be of an acceptable scientific standard, for reasons outlined above.

# Response

**Published:** 2025-09-02

**Authors:** Bruce Abugri

**Affiliations:** Health Professions Education, University for Development Studies, Tamale, Northern Region, Ghana

**Reviewer Comment ** “The manuscript addresses a relevant and timely topic. However, the presentation of the manuscript would benefit from greater clarity and coherence in both writing and structure. For example, the introduction includes content that would be more appropriately placed in the discussion section. Although references are included, the theoretical framework could be strengthened by incorporating more recent and specific literature on similar studies conducted in low-resource settings.”

**Response:** We appreciate this thoughtful feedback. In the revised manuscript, the introduction has been streamlined to reduce redundancy and improve coherence. Overlapping content on the history and benefits of PBL was condensed into concise paragraphs, and the theoretical and conceptual framework has been relocated to the Methods section, where it is more appropriately discussed. In addition, we have incorporated more recent literature on PBL in low-resource and LMIC settings (e.g., Al-Drees et al., 2015; AlHaqwi et al., 2015; Tadesse et al., 2022; Errabti et al., 2024). This strengthens the rationale and situates the protocol within the current global evidence base while highlighting the paucity of research in African first-year medical education contexts. Reviewer Comment: “The proposed design is interesting, but the study has not been carried out. It is a theoretical research proposal. The lack of actual implementation weakens the impact of the article. Readers… expect access to concrete data.” Response: We agree with the reviewer’s observation. To address this, we have clarified throughout the manuscript that this is a study protocol rather than a completed study. We have adjusted the title, abstract, and conclusion to emphasize that the work outlines the planned methodology and anticipated contributions, while acknowledging that data collection and analysis have not yet been undertaken.

**Reviewer Comment ** “The methodology… lacks sufficient operational details to allow replication. It would be necessary to include more specific information regarding variables, instruments, procedures, and contextual conditions **.”**

**Response**: We have expanded the Methods section to provide additional operational detail. Specifically, we now describe the sample size determination process using G*Power 3.1, specify the expected number of eligible participants (110 first-year students), and explain the census approach. We also provide clearer descriptions of the Critical Thinking Questionnaire (Kobylarek et al., 2022) and Interpersonal Communication Competence Scale (Rubin & Martin, 1994; Hande et al., 2015; Jdaitawi, 2020), as well as the Likert-scale measure of student satisfaction (Al-Drees et al., 2015; AlHaqwi et al., 2015; Tadesse et al., 2022; Errabti et al., 2024). Details on tutor training, fidelity monitoring, and data collection procedures have been elaborated to enhance replicability.

**Reviewer Comment ** “The article does not include any statistical analysis or data interpretation, as it is solely a research proposal.”

**Response:** We acknowledge this point. As this is a protocol manuscript, we have clarified that no empirical results are yet available. However, we have expanded the Data Analysis subsection to specify the planned statistical tests (paired t-tests, ANOVA, regression, and effect size calculations) and to explain the conditions under which these analyses will be applied.

**Reviewer Comment ** “The limitations of the study are not explicitly discussed.The primary limitation is that the study remains a theoretical proposal.The proposed sample size approximately 100 students would be relatively small for generalizable conclusions, especially considering contextual variables.”

**Response:** We appreciate this important observation. In response, we have added a dedicated Limitations subsection. This explicitly acknowledges that the primary limitation is the protocol nature of the work, with no data available at present. We also note that while the census approach ensures coverage of all eligible students, the cohort size may constrain external generalizability due to contextual and resource-related factors. These limitations are now clearly stated to provide readers with appropriate expectations.

**Reviewer Comment ** “No empirical data are presented… so there is no information available to verify results.”

**Response:** We agree. We have clarified in the Data availability section and reiterated in the Abstract and Conclusion that no data are associated with this protocol at this stage. We emphasize that findings will be reported in subsequent publications once data collection and analysis are complete.

**Reviewer Comment ** “Any form of empirical analysis does not support the conclusions… the final statements are speculative in nature.” **R**

**esponse:** We thank the reviewer for this observation. In the revised manuscript, the Discussion and Conclusion have been reframed in the future tense to highlight anticipated contributions rather than presenting outcomes. Statements have been modified to emphasize that the study is designed to generate evidence, and conclusions are now offered as expectations rather than definitive findings.

# Reviewer report: Reviewer response for version 1

**DOI:** 10.21956/mep.22606.r42497

**Published:** 2025-08-30

**Authors:** Michael R. L. Odell

**Affiliations:** 1 The University of Texas at Tyler, Tyler, Texas, USA

This article presents a study protocol proposing a longitudinal pre-test/post-test design to assess the impact of Problem-Based Learning (PBL) on critical thinking, communication skills, and student satisfaction among first-year medical students at the University for Development Studies in Ghana. The manuscript is comprehensive in scope and includes detailed background, theoretical frameworks, methodological plans, and ethical considerations. However, it is important to note that the article is a proposal rather than a completed research study. No data has yet been collected or analyzed, and results are not reported.

 The rationale for the study is generally well described. The authors effectively identify both a contextual gap (lack of data from African settings) and a conceptual gap (limited research on first-year medical students). The literature review is thorough and supports the need for the proposed study. However, the manuscript could benefit from more concisely summarizing the research gap and objective at the end of the introduction to better orient the reader. Some repetition across the background, problem statement, and significance sections may reduce clarity.

 The study design, a longitudinal pre-test/post-test format using validated instruments, is appropriate for the stated research questions. The plan to use the Critical Thinking Questionnaire (CThQ), Interpersonal Communication Competency Scale (ICCS), and a Likert-scale measure of satisfaction is sound. The conceptual framework is clearly aligned with the theoretical underpinnings of PBL, including constructivist, self-directed, and social learning theories. The use of paired t-tests, ANOVA, and regression analyses is appropriate given the objectives.

 In terms of methodological detail, the protocol provides useful information about participant selection, inclusion criteria, sampling strategy, and ethical safeguards. However, several areas require additional specificity to ensure replicability. The manuscript does not provide sufficient detail about how PBL facilitators will be trained or how fidelity to the intervention will be monitored. Similarly, although a census approach is planned, there is no power calculation or justification for the sample size, which weakens confidence in the study’s capacity to detect meaningful effects.

 The article does not present any datasets, which is expected given that it is a study protocol. It is important, however, to clearly label the article as a protocol in all relevant sections to avoid confusion. The authors note that no data are associated with the article at this time, which is appropriate for its current stage.

 Overall, the manuscript is not yet suitable for review as a completed research study but is appropriate for indexing as a study protocol if that is permitted by the journal. The authors should consider waiting to submit the study for full peer review until after data collection and analysis are complete. In the meantime, the following improvements would strengthen the current protocol: reducing redundancy in the introduction, adding detail about facilitator training and intervention fidelity, and providing justification for the sample size.

 This protocol addresses a significant gap in the literature and has the potential to contribute valuable data on the effects of PBL in low-resource, early medical education settings. Once implemented and analyzed, the findings could inform both curriculum reform and instructional practices in Ghana and similar contexts.

Is the study design appropriate for the research question?

Yes

Is the rationale for, and objectives of, the study clearly described?

Yes

Are sufficient details of the methods provided to allow replication by others?

Partly

Are the datasets clearly presented in a useable and accessible format?

Not applicable

Reviewer Expertise:

My area of Expertise is STEM Education P-16.  I am also considered by many to be a PBL expert.

I confirm that I have read this submission and believe that I have an appropriate level of expertise to confirm that it is of an acceptable scientific standard, however I have significant reservations, as outlined above.

# Response

**Published:** 2025-09-02

**Authors:** Bruce Abugri

**Affiliations:** Health Professions Education, University for Development Studies, Tamale, Northern Region, Ghana

Response

**Reviewer Comment 1** “The manuscript could benefit from more concisely summarizing the research gap and objective at the end of the introduction to better orient the reader. Some repetition across the background, problem statement, and significance sections may reduce clarity.”

 Author Response: We appreciate the reviewer’s careful reading and constructive suggestion. In revising the manuscript, we streamlined the Introduction to reduce redundancy and improve clarity. Specifically: We condensed the historical overview of medical education into one paragraph that briefly traces the shift from apprenticeship to competency-based, student-centred approaches (Cooke et al., 2010; Harden, 2020; Schuwirth & van der Vleuten, 2020). We retained a single concise definition and origin of PBL (Barrows, 1986; Neufeld & Barrows, 1974), and removed overlapping descriptions of its benefits and historical development. We shortened the discussion of PBL advantages and criticisms into balanced, non-repetitive statements, while preserving key citations (Dolmans et al., 2016; Neville, 2009; Manuaba et al., 2022). We highlighted the global evidence base by focusing on the most recent and comprehensive reviews (Trullàs et al., 2022; Sayyah et al., 2017), while moving older or duplicative references to the Discussion. We retained only one mention of PBL’s implementation at UDS in 2007 (Amoako-Sakyi & Amonoo-Kuofi, 2015; Mogre & Amalba, 2015) and removed repetitions. We relocated the theoretical and conceptual framework to the Methods section, where it more appropriately supports study design and instrument selection. Finally, we closed the Introduction with a concise summary paragraph that clearly articulates the research gap and objective: namely, the lack of evidence on the effects of PBL among first-year medical students in low-resource African settings, and the aim of this protocol to evaluate its influence on critical thinking, communication competence, and satisfaction at UDS.

**Reviewer Comment 2** “Similarly, although a census approach is planned, there is no power calculation or justification for the sample size, which weakens confidence in the study’s capacity to detect meaningful effects.”

 Author Response: We thank the reviewer for this important observation. In the revised manuscript, we have added a detailed sample size justification supported by an a priori power analysis. Using G*Power 3.1 for a two-tailed paired t test with α = .05 and power = 0.80, the analysis indicated that 34 participants are required to detect a medium effect size (d = 0.50), 51 participants for d = 0.40, and 90 participants for d = 0.30. As the expected cohort size for the 2024/2025 academic year is 110 students, the planned census approach is anticipated to exceed the minimum requirements, thereby ensuring adequate statistical power to detect small-to-moderate effect sizes.
